# Association between Internet use and depressive symptoms among older adults in two regions of Myanmar: a cross-sectional study

**DOI:** 10.1186/s12877-024-04729-4

**Published:** 2024-02-12

**Authors:** Yuri Sasaki, Yugo Shobugawa, Ikuma Nozaki, Daisuke Takagi, Yuiko Nagamine, Yuki Shirakura, Kay Thi Lwin, Poe Ei Zin, Thae Zarchi Bo, Tomofumi Sone, Hla Hla Win

**Affiliations:** 1 Department of Public Health Policy, National Institute of Public Health, Wako City, 351-0197 Japan; 2 Niigata University Graduate School of Medical and Dental Sciences, Niigata City, 951-8510 Japan; 3 National Center for Global Health and Medicine, Bureau of International Health Cooperation, Tokyo, 162-8655 Japan; 4 Department of Health and Social Behavior, Graduate School of Medicine, The University of Tokyo, Tokyo, 113-0033 Japan; 5 Department of Global Health Promotion, Tokyo Medical and Dental University, Tokyo, 113-8510 Japan; 6 Center for Preventive Medical Science, Chiba University, Chiba, Japan; 7 Department of Preventive and Social Medicine, University of Medicine 1, Yangon, 245 Myanmar; 8 National Institute of Public Health, Wako City, 351-0197 Japan

**Keywords:** Depressive symptoms, Geriatric depression scale (GDS), Internet use, Myanmar, Subjective economic status

## Abstract

**Background:**

Internet use has both positive and negative effects on mental health. However, few studies have examined the association between internet use and mental health among older adults in developing countries. This study aimed to investigate the association between Internet use and depressive symptoms among older adults in two regions of Myanmar.

**Methods:**

Data based on a visit to 1,200 older adults in urban and rural Myanmar were obtained through stratified random sampling using the cross-sectional baseline survey of the longitudinal study titled “Healthy and Active Aging in Myanmar.”

Our analysis included 1,186 participants. The dependent variable was depressive symptoms, and the 15-item version of the Geriatric Depression Scale (GDS) was used as a continuous variable; the higher the score, the more likely a person was to be depressed. Internet use (one of the questions about household property ownership) was used as an independent variable. After confirming the absence of multicollinearity, we adjusted for age, gender, educational background, activities of daily living, residential area, and frequency of meeting friends, and stratified by subjective economic status (above or below average). We also examined the interaction between internet use and subjective economic status. A linear regression analysis was performed.

**Results:**

Among the 1,186 participants included in the analysis (women: 59.5%; median age: 68 years old), 202 (17.0%) were Internet users (95% Confidential Interval [CI]: 0.15, 0.19), and they had significantly lower GDS scores than the participants who did not use the Internet (B: -1.59, 95% CI: -2.04, -1.13).GDS showed a negative association with Internet use even in the multivariate analysis (B: -0.95, 95% CI: -1.41, -0.50). However, the interaction term for GDS between Internet use and subjective economic status was not significantly associated (B: 0.43, 95% CI: -1.11, 1.98).

**Conclusions:**

Internet use and depressive symptoms were associated especially among the older adults. However, there were no significant interaction between Internet use and subjective economic status for GDS.

## Background

Worldwide, new digital technologies such as the Internet have changed how people connect and interact. The transition to web-enabled internet is occurring at a rapid rate, with an estimated reach of 4.9 billion individuals worldwide in 2021, up from 4.6 billion in the previous year [1].

Several studies have claimed that Internet use can influence the health of older adults [2, 3]. A literature review on social media use among older adults indicates that online communities are suitable for providing and receiving social support when people are confronted with difficult life situations, regardless of their geographical location or time. Further positive consequences include overcoming loneliness, relieving stress, and raising feelings of control and self-efficacy [3]. Social media use can help individuals organize their work, increase availability, and stay connected with family and friends [4]. Studies in China have indicated a significant positive relationship between social media use and the health of older adults [2, 5]; the correlation was more significant for their mental health than their physical health [2]. Data from a nationally representative sample of US Medicare beneficiaries aged 65 years and older also indicated that depressive and anxiety symptoms were negatively associated with Internet use [6].

During the coronavirus disease 2019 (COVID-19) outbreak, Internet use also drew increasing attention for mental health care, especially among older adults who had been advised to avoid social activities. According to a scoping review conducted in European countries, the United States and Hong Kong, Internet use for communication was associated with improved mental health among older adults during the pandemic [7].

However, Internet use is also known to have negative consequences. It may distract individuals from communicating face-to-face with other members of society [8]. Research on the effects of Internet use on the mental health of older Chinese adults showed that Internet use had increased the incidence of depressive symptoms.

In Hong Kong, social media is a double-edged sword for mental health, and its roles varied across age groups during the COVID-19 pandemic; social media use had a significantly negative direct effect on depressive symptoms among older adults, but the effect mediated by posttraumatic stress disorder was significantly positive both among older and younger populations [9].

Considering the increasing prevalence of Internet usage, examining whether Internet use positively or negatively impacts the mental health of older adults is imperative. In addition, because COVID-19 hinders direct connections with others, verifying the association between indirect connections via Internet use and mental health among older adults who are at a high risk of infectious diseases is important. In particular, research on older adults in low-income countries is essential because their living conditions, life situations, and Internet use will likely differ from those of the younger generation and those in high-income countries. To the best of our knowledge, no studies conducted in Myanmar explore the association between Internet use and health. However, since the military regime took control in Myanmar on February 1, 2021, there have been reports of the regime implementing restrictions on disseminating critical information. This restriction includes the imposition of increasingly severe blockades on the Internet and social media platforms [10]. Although this study utilizes data collected before the military regime, we investigated the association between internet use and depressive symptoms among older adults in two regions of Myanmar.

## Methods

### Study design and participants

This study used survey data on healthy and active aging conducted in the Yangon and Bago regions of Myanmar in 2018. The participants were independent older adults aged 60 years living in the region, and follow-up surveys are currently underway.

The sample size was calculated based on the WHO STEPS Surveillance Manual 2017 [11]; since 1200 was the approximate ideal number, face-to-face interviews were conducted with 600 older adults each from Yangon (urban area) and Bago (rural area). Because we planned to continue collecting samples up to the target number, we did not adjust for a certain expected response rate in this survey. Multi-stage random sampling was conducted in the two regions. Further details can be found in the previous studies [12–14].

Trained surveyors visited 1083 people in Yangon, 610 were at home, of whom 6 refused to cooperate with the survey, and 4 who were bedridden and had severe dementia were excluded. Thus, the response rate was 98.4% in Yangon. In Bago, 1044 individuals were visited, and 694 were at home, of whom no one declined the survey. Nonetheless, 94 were excluded because they were bedridden or had severe dementia, resulting in a response rate of 86.5% in Bago. A total of 600 individuals each from the Yangon and Bago regions were surveyed [12]. For the analysis, we utilized data from 1,186 participants. Those missing data on the 15-item Geriatric Depression Scale (GDS) and Internet use were excluded from the analysis.

### Dependent variable

Depressive symptoms were assessed using a validated GDS-15 [15–19]. Studies evaluating the psychometric properties of GDS-15 have demonstrated high sensitivity and specificity when compared to the gold standard assessment through clinical evaluation using Structured Clinical Interviews for DSM-IV [16, 20]. The GDS scores range from 0 to 15 points, with higher scores indicating a more depressive state [13]. Cronbach’s alpha in our study was 0.76.

### Independent variables

Whether or not a participant was an Internet user was used as the independent variable. Participants were asked about their household assets, one of which was whether they used the Internet. Participants who used the Internet were defined as Internet users.

#### Confounding variables

The following variables that varied the regression coefficient of the exposure factor by 10% or more, and were statistically significantly associated with depressive symptoms in univariate analyses or significantly associated with depressive symptoms in previous studies [13, 21, 22] were entered into the multivariate models: age (60–74 years old or 75 or older); sex (male or female); educational level (no school, monastic, some/all primary school, middle/high school or higher); activity of daily living (ADL) (independence or not); residential area (Yangon or Bago); frequency of meeting friends (hardly/non, less than once a week, or once a week or more); and subjective economic status (difficult/very difficult or average or higher). In our study, the reference variables for each variable were as follows: age (60–74 years old), sex (men); educational level (no school), ADL (independence), residential area (Yangon), frequency of meeting friends (hardly/non), and subjective economic status (difficult/very difficult). As our previous research found that subjective economic status was more likely to be associated with depressive symptoms than objective economic status among older adults in Myanmar, we employed subjective economic status in the present study [13].

### Statistical analysis

We calculated the rates for each category of socio-demographic variables. The skewness or kurtosis test tested the assumption of normality of GDS, and the normality hypothesis was rejected (*p* < 0.01). The Wilcoxon rank-sum and chi-square tests were used. As the residuals of GDS were normally distributed, and equal variances and independence were confirmed in each variable, outliers were not excluded, and linear regression analysis was conducted to examine whether Internet use was associated with depressive symptoms. GDS were entered as continuous variables. To explore our hypothesis that subjective economic status moderates the association between Internet use and depressive symptoms, we included the interaction effect between Internet use and subjective economic status in the multivariate model. The margins plot visually represented this interaction term to enhance its visibility. The subjective economic status of participants was assessed by asking: “Which of the following best describes your current financial situation in light of general economic conditions?” The participants were asked to select from the following five options: 1. very difficult, 2. difficult, 3. average, 4. comfortable, and 5. very comfortable. A small number of respondents reported that their subjective economic status was “very difficult” (*n* = 31) and “very comfortable” (*n* = 5); therefore, participants were categorized as having “average or more” (answering 3, 4, or 5) or “difficult or very difficult” (answering 1 or 2) perceived economic status following a previous study that dichotomized subjective economic status [13].

The multivariate adjusted results were expressed as non-standardized coefficients (B) with 95% confidence interval (CI). We used STATA14 to perform all statistical analyses, and the statistical significance level was set at *p* < 0.05.

## Results

### Characteristics of internet users and non-users

Table 1 describes the characteristics of the participant divided into internet users and non-users. Among the 1,186 participants included in the analysis (women: 59.5%; median age: 68 years old), 202 (17.0%) were Internet users (95% CI: 0.15, 0.19). Compared with participants who did not use the Internet, Internet users had significantly lower median GDS scores (4 vs. 3 scores, *p* < 0.001), higher “excellent” or “good” self-rated health (27.7% vs. 38.1%, *p* = 0.003), and middle school or higher education (26.5% vs. 60.4%, *p* < 0.001). In addition, they mostly lived in the urban area (Yangon) (42.4% vs. 87.6%, *p* < 0.001) and lower levels of “meeting with friends or acquaintances once a week or more” (72.9% vs. 43.6%, *p* < 0.001) and lower subjective economic status with a “difficult” or “very difficult” rating (23.4% vs. 6.9%, *p* < 0.001).

**Table 1:** Comparisons of demographic characteristics between Internet uses and non-users (*N* = 1,186^*^)

Internet use		Users (*n* = 202)	%	Non-users (*n* = 984)	%	*p*-value
Depressive symptoms	Median GDS score(IQR)	3(2–4)		4(3–7)		< 0.001^a^
Self-rated health	Excellent/Good	77	38.10%	273	27.70%	0.003^b^
	Fair/Poor	125	61.90%	711	72.30%	
Age	60–74	151	74.80%	744	75.60%	0.796^b^
	75 or more	51	25.20%	240	24.40%	
Sex	Male	85	42.10%	395	40.10%	0.609^b^
	Female	117	57.90%	589	59.90%	
Education	No school	11	5.40%	91	9.20%	< 0.001^b^
	Monastic school	26	12.90%	260	26.40%	
	Some/Finished primary school	43	21.30%	372	37.80%	
	Middle school or higher	122	60.40%	261	26.50%	
Activity of daily living	Not independent	23	11.40%	143	14.50%	0.24^b^
	Independent	179	88.60%	841	85.50%	
Region	Yangon	177	87.60%	417	42.40%	< 0.001^b^
	Bago	25	12.40%	567	57.60%	
Meeting friends/acquaintances	Hardly/None	56	27.70%	140	14.20%	< 0.001^b^
	Less than once a week	58	28.70%	127	12.90%	
	Once a week or more	88	43.60%	717	72.90%	
Subjective economic status	Difficult/Very difficult	14	6.90%	230	23.40%	< 0.001^b^
	Average or higher	188	93.10%	754	76.60%	

*IQR* Interquartile Range

^a^*p*-value for Wilcoxon rank sum test

^b^*p*-value for Chi-square test

^*^14 participants with missing data on the 15-item Geriatric Depression Scale (GDS) and Internet use were excluded

### Bivariate associations between demographic characteristics and GDS scores

Table 2 shows the bivariate associations between demographic and depressive symptoms. Of the 1,186 participants included in the analysis, GDS scores were negatively associated with internet use (B: -1.59, 95% CI: -2.04, -1.13). Most demographic variables except age, were significantly associated with GDS scores.

**Table 2:** Bivariate associations between demographic characteristics and depressive symptoms (*N* = 1,186)

	GDS (Linear regression)	B	SE	95% CI		*p*-value	Adj R-squared
Internet	Do not use	ref					
	Use	-1.59	0.23	-2.04	-1.13	< 0.01	0.04
Age	60–74	ref					
	75 or more	0.35	0.21	-0.05	0.76	0.09	0.002
Sex	Male	ref					
	Female	1.04	0.18	0.69	1.39	< 0.01	0.03
Education	No school	ref					
	Monastic school	-0.82	0.34	-1.50	-0.15	0.02	0.05
	Some/Finished primary school	-1.19	0.33	-1.84	-0.54	< 0.01	
	Middle school or higher	-2.23	0.33	-2.88	-1.57	< 0.01	
ADL	Not independent	ref					
	Independent	-1.13	0.25	-1.63	-0.63	< 0.01	0.02
Region	Yangon	ref					
	Bago	0.88	0.18	0.54	1.23	< 0.01	0.02
Meeting friends/acquaintances	Hardly/None	ref					
	Less than once a week	-0.84	0.31	-1.45	-0.22	< 0.01	0.01
	Once a week or more	-0.22	0.24	-0.70	0.26	0.37	
Subjective economic status	Difficult/Very difficult	ref					
	Average or higher	-2.95	0.2	-3.35	-2.55	< 0.01	0.15

Results of bivariate analysis in which each the independent variable is entered into each model

*ADL* Activity of Daily Living, *B* Unstandardized Regression Coefficient, *CI* Confidence Interval, *GDS* Geriatric Depression Scale, *SE* Standard Error

### Multivariate adjusted association between Internet use and GDS scores

Table 3 shows the multivariate-adjusted association between Internet use and depressive symptoms. Of the 1,186 participants included in the analysis, GDS were negatively associated with internet use even after adjusting for confounding variables (B: -0.95, 95% CI: -1.41, -0.50). Average or higher subjective economic status was also significantly negatively associated with GDS, with the point estimate being the largest of the variables (B: -2.61, 95% CI: -3.01, -2.21).

**Table 3:** Multivariate adjusted association between depressive symptoms and Internet use (*N* = 1,186)

	GDS (Linear regression)	B	SE	95% CI		*p*-value
Internet	Do not use	ref				
	Use	-0.95	0.23	-1.4	-0.5	< 0.01
Age	60–74	ref				
	75 or more	0.06	0.19	-0.3	0.44	0.74
Sex	Male	ref				
	Female	0.77	0.17	0.43	1.1	< 0.01
Education	No school	ref				
	Monastic school	-0.24	0.32	-0.9	0.39	0.45
	Some/Finished primary school	-0.4	0.31	-1	0.21	0.2
	Middle school or higher	-0.7	0.34	-1.4	-0.04	0.04
ADL	Not independent	ref				
	Independent	-0.75	0.24	-1.2	-0.29	< 0.01
Region	Yangon	ref				
	Bago	0.14	0.19	-0.2	0.52	0.44
Meeting friends/acquaintances	Hardly/None	ref				
	Less than once a week	-0.51	0.29	-1.1	0.05	0.08
	Once a week or more	-0.41	0.23	-0.9	0.05	0.08
Subjective economic status	Difficult/Very difficult	ref				
	Average or higher	-2.61	0.2	-3	-2.21	< 0.01
					Adj R-squared = 0.20	

*ADL* Activity of Daily Living, *B* Unstandardized Regression Coefficient, *CI* Confidence Interval, *GDS* Geriatric Depression Scale, *SE* Standard Error

**Fig. 1:**
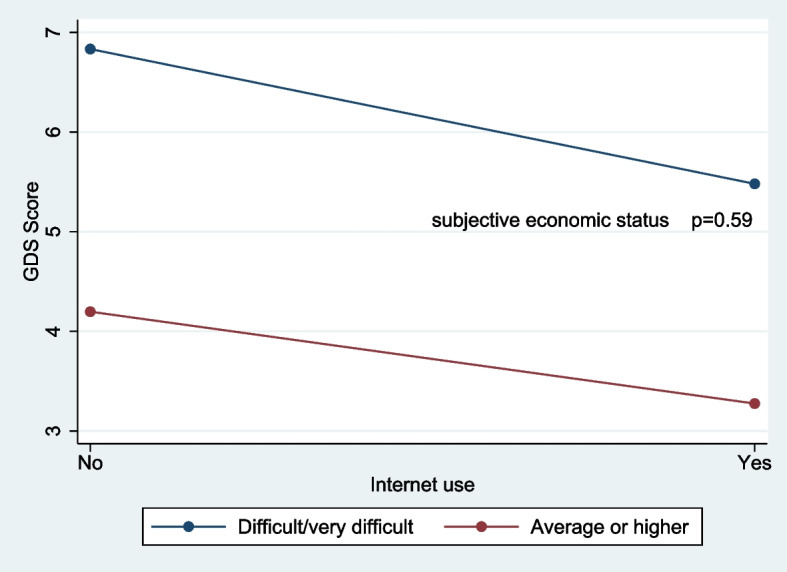
Interaction for GDS scores between Internet use and subjective economic status

A model including an interaction term for GDS between Internet use and subjective economic status, the interaction term for GDS was not significantly associated (B: 0.43, 95% CI: -1.11, 1.98) (Table 4). This indicates no significant slope differences in GDS and Internet use between individuals with average or higher subjective economic status and those with difficult or very difficult subjective economic status (*p* = 0.59) (Fig. 1).

**Table 4:** Multivariate adjusted association between depressive symptoms and Internet use with interaction term between Internet use and subjective economic status

*N* = 1,186		B	SE	95% CI		*p*-value
Internet	Do not use	ref				
	Use	-1.35	0.76	-2.85	0.15	0.08
Age	60–74	ref				
	75 or more	0.06	0.19	-0.32	0.44	0.75
Sex	Male	ref				
	Female	0.77	0.17	0.43	1.1	< 0.01
Education	No school	ref				
	Monastic school	-0.25	0.32	-0.89	0.38	0.44
	Some/Finished primary school	-0.41	0.31	-1.02	0.2	0.19
	Middle school or higher	-0.71	0.34	-1.37	-0.05	0.04
ADL	Not independent	ref				
	Independent	-0.75	0.24	-1.21	-0.29	< 0.01
Region	Yangon	ref				
	Bago	0.14	0.19	-0.23	0.51	0.46
Meeting friends/acquaintances	Hardly/None	ref				
	Less than once a week	-0.51	0.29	-1.08	0.05	0.07
	Once a week or more	-0.41	0.23	-0.86	0.04	0.08
Subjective economic status	Difficult/Very difficult	ref				
	Average or higher	-2.64	0.21	-3.05	-2.22	< 0.01
Internet use *subjective economic status		0.43	0.79	-1.11	1.98	0.59
					Adj R-squared = 0.20	

*ADL* Activity of Daily Living, *B* Unstandardized Regression Coefficient, *CI* Confidence Interval, *GDS* Geriatric Depression Scale, *SE* Standard Error

*Multiplication

## Discussion

To the best of our knowledge, this is the first study to investigate whether Internet use is positively or negatively associated with depressive symptoms among older adults in a low income country, using data from urban and rural areas. Moreover, we conducted an interaction analysis between Internet use and subjective economic status. Overall, the model with adjusted potential confounding factors suggested that older adults who used the Internet were more likely to have lower GDS than those who did not. When examining the interaction effect, there were no significant interaction between Internet use and subjective economic status for GDS.

We found that Internet use among older adults in the two regions of Myanmar was still less common. Although it is thought that the rate was even lower after the two regions became military regime on February 1, 2021, the Internet use ratio of 17.5% in this study was low compared to the full data, which included people of other age groups in Myanmar (43.3% in 2021) [23]. There are various possible reasons older adults were less likely to use the internet in this study. The popularity of Internet use is significantly affected by the level of economic development [24]. In Myanmar, older adults are generally poor. According to the first national research on the situation of older adults in Myanmar in 2012, one-third of older adults live in homes without electricity and over half lack running water, and these situations are particularly common in rural areas [25]. Because of the low economic status of older adults in Myanmar, the number of older adults using the Internet might be small. In addition, invisible obstacles might impede Internet adoption among older age groups. In a study in Israel, older adults often did not fully understand the specific benefits of using online health services, which led to them being less likely to use it [26]. They often struggle with reduced reactivity, which made it harder to keep up with fast-paced technologies [27]. According to Eurostat, 9% of adults in the EU aged 75 or over had severe visual impairments, and 18% had severe hearing limitations in [27]. US statistics also indicated that 23% of older adults had a physical or health condition that made reading difficult or challenging [28]. This may also be the reason for the low rate of Internet use among older adults in this study.

Consistent with the literature from middle- and high-income countries [5, 6, 29–31], our study confirmed that older adults in Myanmar who used the Internet were less likely to be at higher risk of depressive symptoms, even after adjusting for some potential confounding variables, including frequency of meeting friends. Older adults in Myanmar may have used the Internet to contact family and friends to buffer isolation and reduce depressive symptoms, but it may not have reduced the frequency of meeting friends in Myanmar settings. According to a review of the impact of loneliness on the digital world using a theoretical model, the Internet was a useful tool for reducing loneliness when used as a station on the route to enhancing existing relationships and forging new social connections [32]. However, when it was used to escape the social world, feelings of loneliness increased [32]. In the case of older adults in Myanmar, Internet use seemed to function in the former capacity because, culturally, older adults in Myanmar typically hold high status and respect in their families, and family is essential [33]. Moreover, the notion of family extends well beyond the nuclear family in Myanmar society [34].

Although some studies conducted in middle- and high-income countries have identified an association between Internet use and depressive symptoms, no study conducted an interaction analysis between Internet use and subjective economic status, which provides an in-depth examination of the effect of Internet use on depressive symptoms among older adults. However, we found no significant interaction effect. It is possible to interpret that even older adults with poor subjective economic status are less prone to depressive symptoms as long as they are able to use the Internet. As there also may be a mediating effect rather than an interaction effect on depressive symptoms, we will examine which is appropriate in the future.

This study has several limitations [13]. First, it is undeniable that the face-to-face interviews may have created a social desirability bias. This may have resulted in misreporting of depressive symptoms. Second, in this study, the measurement of depressive symptoms was based solely on the GDS score, and clinical diagnoses were not evaluated in determining depressive symptoms. Therefore, the results of this study do not necessarily apply to the diagnosis of clinical depressive symptoms. However, the GDS is a widely used and validated instrument for assessing depressive symptoms [15–17]. Third, our estimates were based on a relatively small number of participants with difficult or very difficult subjective economic status among Internet users. There was a possibility that individual differences could affect the results. Fourth, Myanmar consists of seven regions and seven states, and the findings of this study cannot be generalized to Myanmar outside of Yangon and Bago. Ideally, this study should be extended to include surrounding areas in the future. Fifth, due to the cross-sectional design, it is undeniable that reverse causality may have occurred.

## Conclusions

The proportion of older adults in Myanmar who use the Internet remains very low. However, interaction using the Internet among older adults should be promoted, since Internet use and depressive symptoms were negatively associated in two urban and rural areas of Myanmar. Also, we cannot deny the possibility that Internet use may have moderated the effect of depressive symptoms even among those with poor subjective economic status.

## Data Availability

The datasets generated and/or analysed during the current study are not publicly available due an ethical restriction on sharing a de-identified data set by an ethics committee, but are available from the corresponding author on reasonable request.

## Abbreviations

ADL: Activity of daily living
CI: Confidence interval
GDS: Geriatric depression scale
WHO: World Health Organization

## References

[1] Internet usage worldwide-statistics & facts. https://www.statista.com/topics/1145/internet-usage-worldwide/. Accessed 3 Aug 2022.

[2] Fu L, Xie Y (2021). The effects of social media use on the health of older adults: an empirical analysis based on 2017 Chinese general social survey. Healthcare (Basel).

[3] Leist AK (2013). Social media use of older adults: a mini-review. Gerontology.

[4] Busch PA, Hausvik GI, Ropstad OK, Pettersen D (2021). Smartphone usage among older adults. Comput Hum Behav.

[5] Wang Y, Zhang H, Feng T, Wang H (2019). Does internet use affect levels of depression among older adults in China? A propensity score matching approach. BMC Public Health.

[6] Choi NG, Dinitto DM (2013). Internet use among older adults: association with health needs, psychological capital, and social capital. J Med Internet Res.

[7] Foong HF, Lim SY, Rokhani FZ, Bagat MF, Abdullah SFZ, Hamid TA, Ahmad SA (2022). For better or for worse? A scoping review of the relationship between internet use and mental health in older adults during the COVID-19 pandemic. Int J Environ Res Public Health.

[8] Tammisalo K, Rotkirch A (2022). Effects of information and communication technology on the quality of family relationships: a systematic review. J Soc Pers Relat.

[9] Yang X, Yip BHK, Mak ADP, Zhang D, Lee EKP, Wong SYS (2021). The differential effects of social media on depressive symptoms and suicidal ideation among the younger and older adult population in Hong Kong during the COVID-19 pandemic: population-based cross-sectional survey study. JMIR Public Health Surveill.

[10] Sharma V, Oo PP, Hollaender J, Scott J (2021). COVID-19 and a coup: blockage of internet and social media access further exacerbate gender-based violence risks for women in Myanmar. BMJ Global Health.

[11] The WHO STEPS Surveillance Manual. https://www.who.int/ncds/surveillance/steps/STEPS_Manual.pdf. Accessed 8 Aug 2022.

[12] Win HH, Nyunt TW, Lwin KT, Zin PE, Nozaki I, Bo TZ, Sasaki Y, Takagi D, Nagamine Y, Shobugawa Y (2020). Cohort profile: healthy and active ageing in Myanmar (JAGES in Myanmar 2018): a prospective population-based cohort study of the long-term care risks and health status of older adults in Myanmar. BMJ Open.

[13] Sasaki Y, Shobugawa Y, Nozaki I, Takagi D, Nagamine Y, Funato M, Chihara Y, Shirakura Y, Lwin KT, Zin PE (2021). Association between depressive symptoms and objective/subjective socioeconomic status among older adults of two regions in Myanmar. PLoS One.

[14] Sasaki Y, Shobugawa Y, Nozaki I, Takagi D, Nagamine Y, Funato M, Chihara Y, Shirakura Y, Lwin KT, Zin PE (2022). Association between happiness and economic status among older adults in two Myanmar regions. Int J Environ Res Public Health.

[15] Yesavage JA, Brink TL, Rose TL, Lum O, Huang V, Adey M, Leirer VO (1982). Development and validation of a geriatric depression screening scale: a preliminary report. J Psychiatr Res.

[16] Nyunt MS, Fones C, Niti M, Ng TP (2009). Criterion-based validity and reliability of the Geriatric Depression Screening Scale (GDS-15) in a large validation sample of community-living Asian older adults. Aging Ment Health.

[17] Zalsman G, Aizenberg D, Sigler M, Nahshoni E, Weizman A (1998). Geriatric depression scale-short form–validity and reliability of the Hebrew version. Clin Gerontol.

[18] Sheikh JI, Yesavage JA (1986). Geriatric Depression Scale (GDS): recent evidence and development of a shorter version. Clin Gerontol.

[19] Wada T, Ishine M, Kita T, Fujisawa M, Matsubayashi K (2003). Depression screening of elderly community-dwelling Japanese. J Am Geriatr Soc.

[20] Rajgopal J, Sanjay TV, Mahajan M (2019). Psychometric properties of the geriatric depression scale (Kannada version): a community-based study. J Geriatr Mental Health.

[21] Sasaki Y, Shobugawa Y, Nozaki I, Takagi D, Nagamine Y, Funato M, Chihara Y, Shirakura Y, Lwin KT, Zin PE (2021). Rural-urban differences in the factors affecting depressive symptoms among older adults of two regions in Myanmar. Int J Environ Res Public Health.

[22] Feng Z, Li Q, Zhou L, Chen Z, Yin W (2021). The relationship between depressive symptoms and activity of daily living disability among the elderly: results from the China Health and Retirement Longitudinal Study (CHARLS). Public Health.

[23] Digital 2021: Myanmar. https://datareportal.com/reports/digital-2021-myanmar. Accessed 14 Aug 2022.

[24] Ochoa-Jimenez D, Moreno-Hurtado C, Ochoa-Moreno W (2018). Economic growth and internet access in developing countries: The case of South America. Proceedings of the 2018 13th Iberian Conference on Information Systems and Technologies (CISTI).

[25] Knodel J. The situation of older persons in Myanmar: results from the 2012 survey of older persons. Yangon and Chiang Mai: HelpAge International; 2013.

[26] Mizrachi Y, Shahrabani S, Nachmani M, Hornik A (2020). Obstacles to using online health services among adults age 50 and up and the role of family support in overcoming them. Israel J Health Policy Res.

[27] Your key to European statistics. https://ec.europa.eu/eurostat/web/digital-economy-and-society/data/database. Accessed 5 Aug 2022.

[28] Smith A (2014). Older adults and technology use.

[29] Li Y, Bai X, Chen H (2022). Social isolation, cognitive function, and depression among Chinese older adults: examining internet use as a predictor and a moderator. Front Public Health.

[30] Wallinheimo AS, Evans SL (2021). More frequent internet use during the COVID-19 Pandemic associates with enhanced quality of life and lower depression scores in middle-aged and older adults. Healthcare (Basel).

[31] Hajek A, König HH. Frequency of contact with friends and relatives via internet and psychosocial factors in middle-aged and older adults during the COVID-19 pandemic. Findings from the German ageing survey. Int J Geriatr Psychiatry. 2022;37(1):10.1002/gps.5623. 10.1002/gps.5623 PMC8646763 34505322

[32] Nowland R, Necka EA, Cacioppo JT (2018). Loneliness and social internet use: pathways to reconnection in a digital world?. Perspect Psychol Sci.

[33] Myanmar (Burmese) Culture. https://culturalatlas.sbs.com.au/myanmar-burmese-culture/burmese-myanmar-culture-family.

[34] Quality of internet use is influenced by economic status. https://borgenproject.org/internet-use-influenced-by-economic-status/.

